# Clofazimine is a broad-spectrum coronavirus inhibitor that antagonizes SARS-CoV-2 replication in primary human cell culture and hamsters

**DOI:** 10.21203/rs.3.rs-86169/v1

**Published:** 2020-10-07

**Authors:** Shuofeng Yuan, Xin Yin, XiangZhi Meng, Jasper Chan, Zi-Wei Ye, Laura Riva, Lars Pache, Chris Chun-Yiu Chan, Pok-Man Lai, Chris Chan, Vincent Poon, Naoko Matsunaga, Yuan Pu, Chun-Kit Yuen, Jianli Cao, Ronghui Liang, Kaiming Tang, Li Sheng, Yushen Du, Wan Xu, Kong-Hung Sze, Jinxia Zhang, Hin Chu, Kin-Hang Kok, Kelvin To, Dong-Yan Jin, Ren Sun, Sumit Chanda, Kwok-Yung Yuen

**Affiliations:** University of Hong Kong; Sanford Burnham Prebys Medical Discovery Institute; University of Hong Kong; The University of Hong Kong; State Key Laboratory of Emerging Infectious Diseases, Carol Yu Centre for Infection, Department of Microbiology, Li Ka Shing Faculty of Medicine, The University of Hong Kong; Sanford Burnham Prebys Medical Discovery Institute; Sanford Burnham Prebys Medical Discovery Institute; University of Hong Kong; University of Hong Kong; The University of Hong Kong; The University of Hong Kong; Sanford Burnham Prebys Medical Discovery Institute; Sanford Burnham Prebys Medical Discovery Institute; University of Hong Kong; The University of Hong Kong; University of Hong Kong; University of Hong Kong; UCLA; Department of Molecular and Medical Pharmacology, David Geffen School of Medicine, University of California at Los Angeles, Los Angeles, California 90095, USA.; University of Hong Kong; University of Hong Kong; Department of microbiology; University of Hong Kong; The University of Hong Kong; The University of Hong Kong; University of Hong Kong; The University of Hong Kong; Sanford Burnham Prebys Medical Discovery Institute; University of Hong Kong

**Keywords:** Clofazimine, coronavirus inhibitor, pan-coronaviral inhibitory activity, COVID-19, coronavirus (CoV) outbreak

## Abstract

COVID-19 pandemic is the third zoonotic coronavirus (CoV) outbreak of the century after severe acute respiratory syndrome (SARS) in 2003 and Middle East respiratory syndrome (MERS) since 2012. Treatment options for CoVs are largely lacking. Here, we show that clofazimine, an anti-leprosy drug with a favorable safety and pharmacokinetics profile, possesses pan-coronaviral inhibitory activity, and can antagonize SARS-CoV-2 replication in multiple *in vitro* systems, including the human embryonic stem cell-derived cardiomyocytes and *ex vivo* lung cultures. The FDA-approved molecule was found to inhibit multiple steps of viral replication, suggesting multiple underlying antiviral mechanisms. In a hamster model of SARS-CoV-2 pathogenesis, prophylactic or therapeutic administration of clofazimine significantly reduced viral load in the lung and fecal viral shedding, and also prevented cytokine storm associated with viral infection. Additionally, clofazimine exhibited synergy when administered with remdesivir. Since clofazimine is orally bioavailable and has a comparatively low manufacturing cost, it is an attractive clinical candidate for outpatient treatment and remdesivir-based combinatorial therapy for hospitalized COVID-19 patients, particularly in developing countries. Taken together, our data provide evidence that clofazimine may have a role in the control of the current pandemic SARS-CoV-2, endemic MERS-CoV in the Middle East, and, possibly most importantly, emerging CoVs of the future.

## Introduction

The current pandemic of novel Coronavirus Disease 2019 (COVID-19) caused by severe acute respiratory syndrome coronavirus 2 (SARS-CoV-2) represents a global public health crisis. SARS-CoV-2 infection in human has a broad clinical spectrum ranging from mild to severe cases, with a mortality rate of ~ 6.4% worldwide^1^. As of September 29, 2020, over 33 million cases had been reported in 235 countries, areas or territories with more than 1 million deaths, whereas a sizable portion of infected but non-symptomatic people with potential of transmissibility was also reported^2^.

The genetically diverse coronavirus (CoV) family, currently composed of four genera (α, β, γ, and δ), infects birds, bats and a variety of mammals^3^. Within a decade, the world’s human population has undergone three major CoV outbreaks. SARS-CoV-1 emerged in Guangdong, China in 2002 and, with the aid of commercial air travel, spread rapidly and globally, causing more than 8,000 cases with 10% mortality^4^. In 2012, MERS-CoV may have evolved to infect humans through bats by way of an intermediate camel host, causing over 1,700 cases with almost 40% mortality, and, like SARS-CoV-1, air travel has fueled global spread to 27 countries^5^.

Currently, there are no widely available specific antiviral therapies for CoV in humans. Remdesivir exhibited pan-coronavirus inhibitory potential^6^, and was recently granted emergency use authorization by the FDA for the treatment of COVID-19 based on the significant reduced time to recovery^7^. However, the therapy is far from optimal, particularly for severe COVID-19 patients, and can only be administered intravenously to hospitalized patients^8,9^ Thus development of additional therapeutic options is urgent, as well as the establishment of combinatorial regimens, such as the triple antiviral combination of interferon beta-1b, lopinavir-ritonavir, and ribavirin, which has been shown to be beneficial in a clinical trial^10^.

In efforts to accelerate the development of novel therapies for COVID-19, we previously profiled a library of known drugs encompassing approximately 12,000 clinical-stage or FDA-approved small molecules^11^. In this study, we focused on the antiviral mechanisms of action and *in vivo* efficacy of clofazimine, an FDA-approved molecule discovered as an anti-tuberculosis drug in 1957 and later used for treatment of leprosy^12^. Treating tuberculosis, clofazimine exhibits a minimum inhibitory concentration of 0.016 μg/ml (equivalent to 33.80 nM). The effective concentration of clofazimine against SARS-CoV-2 (half maximal effective concentration 310 nM) is clinically achievable with standard dosage in patients (peak serum concentration 861 nM)^13^. Here, we report the capability of clofazimine to confer protection against SARS-CoV-2 infection in primary human cell and animal models. Most importantly, clofazimine is affordable by COVID-19 patients in developing countries which may substantially relieve the critical care pressure caused by continuing pandemic^14^.

## Results

### Clofazimine Inhibits SARS-CoV-2 and MERS-CoV Replication in Human Cellular Models

Clofazimine has been found to be well tolerated in humans, showing a desirable safety profile at doses of 200mg/day in human^13^, a C_max_ of > 861 nM, and a selectivity index (CC_50_/EC_50_) around 30~50 against SARS-CoV-2 infection^15^ (Figure 1a). These data suggest that therapeutic dosing of clofazimine may be achievable in patients at concentrations likely to have *in vivo* antiviral activity. Using SARS-CoV-2 infection as a model, we further characterized the antiviral activity of clofazimine in human embryonic stem cell-derived cardiomyocytes that robustly support SARS-CoV-2 replication^16^. Strikingly, and in a dose-dependent manner, clofazimine treatment reduced viral titers in the cell lysate by >3-log10 at a concentration of 10μM when compared with the DMSO control (Figure 1b). Next, we assessed the antiviral activity of clofazimine in an *ex vivo* lung culture system. Donor lung tissue was infected with SARS-CoV-2 for 24 h with drug treatment starting at 2 hours post-inoculation (hpi). Our results revealed that clofazimine potently antagonized viral replication in tissues that reflect the primary site of SARS-CoV-2 replication (Figure 1c). To explore whether clofazimine confers protection against another epidemic CoV, we performed a plaque reduction assay for MERS-CoV. Clofazimine reduced MERS-CoV replication in VeroE6 cells with an EC_50_ of 1.48±0.17 μM (Figure 1d). Immunofluorescence staining for MERS-CoV NP illustrated dramatic suppression of virus infection upon clofazimine treatment (upper panel, Figure 1e), which is supported by the flow cytometry analysis that the percentage of MERS-CoV-infected cells after clofazimine treatment decreased from 44.6% (DMSO) to 23.0% (clofazimine) at 24 hpi in Huh7 cells (lower panel, Figure 1e). Overall, clofazimine exhibited potent broad spectrum anti-CoV, and antagonized SARS-CoV-2 replication in human primary cell and *ex vivo* lung models.

### Clofazimine Interferes with Multiple Steps of Virus Life Cycle

To understand the impact of clofazimine on the virus life cycle, antiviral activity was first evaluated by a time-of-drug addition assay in a single infectious cycle. Treatment with clofazimine during inoculation strongly inhibited SARS-CoV-2 infection, indicating that clofazimine exerts inhibitory effect on viral entry. Intriguingly, clofazimine also blocked SARS-CoV-2 infection at a post-entry step as evidenced by the observed reduction of viral infection when clofazimine was added at 5 hpi (Figure 2a). To further evaluate the impact of clofazimine on viral entry, we employed vesicular stomatitis virus (VSV)-based SARS-CoV-2 Spike (S) pseudotyped virions. Clofazimine treatment dramatically reduced the infectivity of both SARS-CoV-1 S and SARS-CoV-2 S pseudotyped virions in VeroE6 cells. Interestingly, clofazimine did not impact MERS-CoV S pseudotyped virus particles (Figure 2b), and this lack of entry inhibition may contribute to a lower potency observed for MERS-CoV. To confirm whether clofazimine also inhibits post-entry steps of viral replication, we evaluated the impact of clofazimine on viral RNA production by electroporating *in vitro* transcribed viral RNA into VeroE6 cells, which bypasses clofazimine-mediated inhibition on the entry process, and directly measures RNA synthesis (Figure 2c). As expected, remdesivir could effectively reduce the synthesis of negative-stranded RNA in a dose-dependent manner (Figure 2d). Intriguingly, viral RNA levels were also reduced by 1~1.5 logs in the cells treated with clofazimine at concentrations above 5 μM (Figure 2e). However, no significant effect was observed on electroporated GFP mRNA translation (Figure 2f). Collectively, these results demonstrated that clofazimine inhibit multiple steps in SARS-CoV-2 replication by interfering with spike-mediated entry as well as viral RNA replication.

### Transcriptional Analysis of Clofazimine Treatment

To explore what is the impact of clofazimine on the transcriptional response of host cells, we employed RNA-Seq to profile the transcriptomic-wide changes during clofazimine treatment. We found that in human colorectal Caco-2 cells, clofazimine exhibited comparable anti-SARS-CoV-2 potency as that of remdesivir (Figure 3a), which was chosen for the downstream analysis. Transcriptional analysis was performed in Caco-2 cells which were either infected with SARS-CoV-2, treated with clofazimine (10 μM) or both. Principal Component Analysis (PCA) on RNA-Seq results suggested that at 3 hpi, clofazimine treatment (3hpi. CFZ) caused overall transcriptome shift towards mock-infection group when compared with the vehicle control group (3 hpi) (Figure 3b), which is consistent with our data indicating that the drug inhibits viral infection at early time point post infection (Figure 2). At 6 hpi, there were 607 and 448 genes up- and down-regulated by SARS-CoV-2 infection, respectively (FDR<0.05, fold change>2 or <0.5 compared with mock). The RNA level of more than 90% of these genes was reverted by clofazimine treatment, indicating that clofazimine treatment abrogated transcriptomic changes caused by SARS-CoV-2 infection. This is consistent with the PCA plot that treatment with clofazimine at 6 hpi (6 hpi. CFZ) caused a dramatic shift towards mock (Figure 3b, Extended Data Figure 1a). Interestingly, clofazimine treatment in the absence of infection (6h. CFZ) up-regulated genes that were enriched into innate immunity-related pathways, including MAPK, interleukin and TNF responses (Figure 3c and 3d, Extended Data Figure 1b). Particularly, transcription factors critical for immediate-early cellular response, including AP-1, SMAD, MAFF families, were upregulated by clofazimine (Figure 3c). When clofazimine was applied onto infected cells, most of these innate immune pathways were further enriched in upregulated genes (6hpi. CFZ, Figure 3d, Extended Data Figure 1b and 1c). These results suggest that clofazimine rewires the transcriptional landscape to prime the innate immunity-related pathways. While deficient early stage innate immune responses have been attributed to poor disease outcome, additional studies are required to determine if this enhanced antiviral response contributes to the *in vitro* and in *vivo* efficacy of the drug^17–19^.

### Prophylactic and Therapeutic Treatment with Clofazimine Reduces SARS-CoV-2 Disease

Clofazimine is useful for the treatment of disease due to multidrug resistant *Mycobacterium tuberculosis,* as well as leprosy and certain chronic skin diseases^13^.Previous pharmacokinetics studies revealed that clofazimine absorption varies from 45 to 62% following oral administration in leprosy patients. Co-administration of a 200mg dose of clofazimine with food resulted in a C_max_ of 0.41 mg/L (equivalent to 861 nM) with a T_max_ of 8h. Administered in a fasting state, however, the corresponding C_max_ of clofazimine was 30% lower while the time to C_max_ was 12h^20^. Intriguingly, clofazimine exerts anti-inflammatory properties due to the suppression of macrophage activity, which may further mitigate the cytokine storm of SARS-CoV-2 infection in addition to its direct antiviral effects^21^. To determine the *in vivo* antiviral efficacy of clofazimine, we employed a golden Syrian hamster model that serves as a suitable tool to study antiviral effects and disease pathogenesis^22^. Since administration of clofazimine with a high fat meal provides better bioavailability^13^, we delivered the drug through oral route utilizing corn oil as vehicle. 25 mg/kg/day of clofazimine given on 3 consecutive days exhibited no significant observable toxicity to the animals. Remdesivir was included as a positive control drug and dosed at 15 mg/kg/day based on its effective dosage in SARS-CoV-infected mice^6^.

Clofazimine has a relatively long duration of action with the mean elimination half-life approximately 25 days, thus we performed prophylactic treatment of hamsters with clofazimine before intranasally challenged with 10^5^ PFU of SARS-CoV-2 (Figure 4a). Expectedly, the DMSO-treated control hamsters developed the clinical signs of lethargy, hunched back posture, and rapid breathing starting from 2 dpi, whereas the hamsters treated with clofazimine did not develop any clinical signs. At 2 dpi when the viral loads and histopathological changes were expected to be worse, clofazimine decreased virus plaque forming units in lung tissues by ~1 to 1.5 logs (Figure 4b). Consistently, suppression of SARS-CoV-2 viral load in hamster lungs was confirmed in the clofazimine-treated hamsters (Figure 4c). To explore if the presence of clofazimine in the gastrointestinal tract, after intragastric administration, would prevent SARS-CoV-2 shedding, animal feces were collected at 2 dpi for viral RNA detection. Significantly less (p=0.0353) viral copies were detectable in clofazimine-treated group when compared with the DMSO group, indicating its potential to diminish fecal shedding of SARS-CoV-2 (Figure 4d). Increased pro-inflammatory cytokines and chemokines is associated with disease severity of COVID-19 patients. To ascertain if the therapeutic effect of clofazimine alleviates virus-induced cytokine dysregulation, we determined the expression levels of interleukin 6 (IL-6), tumor necrosis factor alpha (TNF-α), and C-C chemokine receptor type 4 (CCR4), which are prognostic markers for severe COVID-19^23^. As shown in Figure 4e, mRNA expression of IL-6 (p=0.0001), TNF-α (p=0.0006), and CCR4 (p=0.0029) were remarkably reduced in the hamsters treated with clofazimine. Previous reports have shown that clofazimine can inhibit lymphocyte function^24^. To explore if this is the case in our animal model, hamster sera were collected at 14dpi for measurement of anti-NP antibody using an ELISA-based enzyme immunoassay. Apparently, similarly high levels of antibody responses were triggered in DMSO and clofazimine groups, indicating insignificant suppression of humoral immune response of B lymphocyte by clofazimine (Figure 4i). Taken together, prophylactic administration of clofazimine conferred protection against SARS-CoV-2 challenge by reducing the virus replication and the associated inflammatory dysregulation.

To recapitulate the scenario that most COVID-19 patients will receive treatment after diagnosis or disease onset, it was of interest to determine whether therapeutic treatment of clofazimine, with the first dosing given 24 h after virus exposure, would also ameliorate SARS-CoV-2 disease. SARS-CoV-2 infected hamsters were given 3 doses in total before being scarified at 4 dpi for lung viral yield detection. Generally, both therapeutic clofazimine and remdesivir suppressed virus lung titers when compared with the DMSO control (Figure 4f and 4g). The diminished clinical signs were also associated with substantially decreased IL-6 protein amount in the clofazimine (p=0.0119) and remdesivir-treated (p=0.0074) hamster sera (Figure 4h), as increased serum IL-6 level has been correlated with respiratory failure and adverse clinical outcome^25^.

As for the severity of lung damage, histological examination of hematoxylin and eosin (H&E) stained lung tissues was performed. Significant amelioration of lung damage was observed after clofazimine treatment (Figure 4j). For prophylactic administration, lung tissues from the DMSO group showed severe bronchiolar cell death with massive cell debris filling the lumen, alveolar wall thickened with alveolar exudation; whereas prophylaxis with clofazimine showed no apparent pathological changes. With therapeutic administration, DMSO-treated lung sections showed large areas of lung consolidation with alveolar infiltration and exudation, while clofazimine treated lungs exhibited a mild degree of alveolar wall thickening and capillary congestion. Generally, prophylactic administration conferred more dramatic improvements of lung pathology when compared with therapeutic administration, which might be attributed to the relatively long T_max_ of clofazimine. Nevertheless, both prophylactic and therapeutic treatment with clofazimine reduced SARS-CoV-2 disease *in vivo.*

### Clofazimine Exhibits Antiviral Synergy with Remdesivir

Since the emergency use authorization by the US FDA, remdesivir is considered the standard of care for the treatment of COVID-19. To understand the impact of combinatorial treatments of remdesivir and clofazimine on SARS-CoV-2 replication, we conducted a matrixed dose response analysis. We found that co-application of clofazimine and remdesivir impacts SARS-CoV-2 replication in a manner that extends beyond the additive combinatorial activity predicted by the Bliss independence model (maximal Bliss Synergy Score of 44.28; Figure 5a, Extended Data Figure 2), and indicates these two drugs harbor a synergistic antiviral relationship. Clofazimine can be safely dosed at 200 mg/day for the treatment of leprosy, which results in average serum concentrations of 1.79 μM, although the bioavailable fraction of the molecule will be a function of plasma protein binding. The addition of 1.25 μM clofazimine in an in vitro cellular assay with a 10% concentration of FBS resulted in a nearly 20-fold decrease in concentrations of remdesivir required to inhibit viral replication by 90% (Figure 5b). Importantly, the combination of drugs did not elicit additional cellular cytotoxicity (Figure 5c).

## Discussion

Clofazimine was first used to treat leprosy in 1969, and gained FDA approval in 1996^26^. It is an orally bioavailable drug that is included in the WHO Model List of Essential Medicines. It is generally well-tolerated, with adverse events that include skin discoloration, ichthyosis, and gastrointestinal intolerance^27^. Besides treating leprosy, clofazimine is an intriguing medication that has implications for multi-drug-resistant tuberculosis (MDR-TB) and extensively drug-resistant tuberculosis (XDR-TB). Showing good safety evidence, clofazimine is a part of WHO group C in terms of the treatment guidelines for MDR-TB^28^. This is supported by clinical trials in China, Bangladesh, and Brazil where patients were receiving clofazimine for 18~21 months at a dose of 100 mg/day^29,30^.

We observed that clofazimine shows pleotropic antiviral activities against SARS-CoV-2, including inhibition of spike-dependent entry. While it has been reported that clofazimine is internalized through endocytosis, further investigation is required to elucidate if the drug directly impinges on endosomal function to inhibit viral entry^31^, and why the leprosy drug selectively blocks SARS-CoV, but not MERS-CoV, entry into cells. Importantly, this drug is a lipophilic rhimophenazine dye which inhibits mycobacteria through intercalation into bacterial DNA, likely inhibiting DNA replication and proliferation^32^. While we observe that clofazimine inhibits the RNA replication of SARS-CoV-2, additional studies are also required to determine if the drug similarly inhibits CoV RNA unwinding or template function.

In SARS-CoV-2 infection, a delayed innate immune response may result in uncontrollable cytokine storm^19,33^. Clofazimine’s effect on rewiring the transcriptional landscape of the cell towards an antiviral status may be important in a disease setting, and understanding the contribution of this activity toward *in vivo* disease amelioration can provide insight towards its potential to improve viral control through enhancement of innate immune activities. Paradoxically, clofazimine has been reported to possess anti-inflammatory activity through the inhibition of macrophage function and T lymphocyte activation and proliferation^34^. Further elucidation of how clofazimine treatment may balance regulation of innate and adaptive immune responses to improve disease outcome will be important to understand its potential clinical efficacy.

In this study, a prophylaxis regimen with three daily doses substantially protected animal from SARS-CoV-2 infection (Figure 4). In contrast to orally bioavailable clofazimine, remdesivir is currently given through intravenous administration, which makes it difficult to provide on an outpatient or prophylactic basis. Moreover, remdesivir requires a complex synthesis process to manufacture, resulting in a high treatment cost (US$520 per vial, or US$3,120 per treatment course) and availability for only several million patients over the next two years^35^. In view of the potentially-long epidemic dynamics and pressures on critical care capacity over the next 5 years, as well as the potential resurgence of SARS-CoV-2 in the future, clofazimine, which only costs US$1.43/100mg tablets, can be considered as one of the potential countermeasures for global control of the COVID-19 pandemic^14^, especially in developing countries. Additionally, co-administration with clofazimine could significantly reduce costs for remdesivir-based treatment of COVID-19, and extend worldwide supplies of remdesivir, and a combinatorial approach can also help mitigate the emergence of drug-resistant viral strains.

Clofazimine, in conjunction with interferon, is currently being evaluated in clinical trials for the treatment of COVID-19 (Trial Number: NCT04465695). The *ex vivo* and *in vivo* efficacy of clofazimine suggests that clinical evaluation of the drug as monotherapy in outpatient setting for treatment of early stage disease, or in combination with remdesivir in hospitalized patients, is critical for establishing its potential as a rapidly scalable treatment option for COVID-19.

## Materials And Methods

### Cells and viruses

Human hepatoma Huh7 (JCRB, 0403) cells and monkey VeroE6 cells (ATCC, CRL-592 1586) were maintained in DMEM culture medium supplemented with 10% heat-inactivated FBS, 50 U/ml penicillin and 50 μg/ml streptomycin. Ventricular cardiomyocyte were differentiated from the human embryonic stem cell HES2 (ESI) maintained in mTeSR1 medium (STEMCELL Technologies)^36^. Briefly, HES2 cells were dissociated with Accutase (Invitrogen) into single cells suspensions on day 0. Cells were seeded on low-attachment culture vessels (Corning) and cultured in mTeSR1 medium supplemented with 40 μg/ml Matrigel, 1 ng/ml BMP4 (Invitrogen) and 10μM Rho kinase inhibitor (ROCK) (R&D) under hypoxic environment with 5% O_2_. From day 1 to 3, cells were cultured in StemPro34 SFM (Invitrogen) with 50 μg/ml ascorbic acid (AA) (Sigma), 2 mM Gluta-MAX (Invitrogen), 10 ng/ml BMP4, and 10 ng/ml human recombinant activin-A (Invitrogen). From day 4 to day 7, 5 μM Wnt inhibitor IWR-1 (Tocris) was added. From day 8 to day 14, cells were cultured under normoxia in RPMI 1640 medium (Invitrogen) supplemented with 2 mM Gluta-MAX, 1×B-27 supplement (Invitrogen) and 50 μg/mL AA. The cells were then dissociated with Accutase and seeded as monolayer in desired culture vessels for 3 days before infections. The SARS-CoV-2 HKU-001a strain (GenBank accession number: MT230904) was isolated from the nasopharyngeal aspirate specimen of a laboratory-confirmed COVID-19 patient in Hong Kong^22^. The SARS-CoV-2, Isolate USA-WA1/2020 was deposited by the Centers for Disease Control and Prevention and obtained through BEI Resources. The MERS-CoV (HCoV-EMC/2012) was a gift from Dr. Ron Fouchier. All experiments involving live SARS-CoV-2 and MERS-CoV followed the approved standard operating procedures of the Biosafety Level 3 facility at the University of Hong Kong as we previously described^37^.

### Antiviral evaluation in human ex vivo lung issues

Human lung tissues for *ex vivo* studies were obtained from patients undergoing surgical operations at Queen Mary Hospital, Hong Kong as previously described^38^. The donors gave written consent as approved by the Institutional Review Board of the University of Hong Kong/Hospital Authority Hong Kong West Cluster (UW13–364). The freshly obtained lung tissues were processed into small rectangular pieces and were rinsed with advanced DMEM/F12 medium (Gibco) supplemented with 2 mM of HEPES (Gibco), I×GlutaMAX (Gibco), 100 U/ml penicillin, and 100 μg/mL streptomycin. The specimens were infected with SARS-CoV-2 HKU-001a with an inoculum of 1×10^6^ PFU/ml at 500 μL per well. After two hours, the inoculum was removed, and the specimens were washed 3 times with PBS. The infected human lung tissues were then cultured in 1 ml of advanced DMEM/F12 medium with 2 mM HEPES (Gibco), 1×GlutaMAX (Gibco), 100 U/mL penicillin, 100 μg/mL streptomycin, 20 μg/mL vancomycin, 20 μg/mL ciprofloxacin, 50 μg/mL amikacin, and 50 μg/mL nystatin. Supernatants were collected at 24 hours post inoculation (hpi) for plaque assays.

### Antiviral assessment in a SARSCoV-2 infected hamster model

Male and female Syrian hamster, aged 6–10 weeks old, were kept in biosafety level 2 housing and given access to standard pellet feed and water ad libitum as we previously described^22^. All experimental protocols were approved by the Animal Ethics Committee in the University of Hong Kong (CULATR) and were performed according to the standard operating procedures of the biosafety level 3 animal facilities (Reference code: CULATR 5370–20). Experimentally, each hamster was intranasally inoculated with 10^5^ PFU of SARS-CoV-2 in 100 μL PBS under intraperitoneal ketamine (200 mg/kg) and xylazine (10 mg/kg) anesthesia. Prophylactic treatment used oral administration of clofazimine given on −3, −2 and −1 dpi (25 mg/kg/day), followed by virus challenge at 0dpi, while therapeutic post-exposure and oral administration of clofazimine were performed on 1, 2, and 3 dpi (25 mg/kg/day) with the first dosage given at 24 hpi. Clofazimine was delivered using corn oil (Sigma-Aldrich, C8267) as vehicle. Remdesivir (15 mg/kg/day, MedChemExpress) was used as a positive control through intraperitoneal injection. One percent DMSO in corn oil was used as a placebo control through oral route. Animals were sacrificed at 2 dpi and 4 dpi for virological and histopathological analyses. Viral yield in the lung tissue homogenates and/or feces were detected by plaque assay and/or qRT-PCR methods. Cytokine and chemokine profile of the hamster lungs were detected by 2^−ΔΔCT^ method using probe-based one step qRT-PCR (Qiagen). ELISA kit was utilized to determine the Interleukin 6 (IL-6) amount in the hamster sera on 4 dpi according to the manufacture’s recommendations (ELISAGenie, HMFI0001). Tissue pathology of infected animals was examined by H&E staining in accordance to the established protocol^39^. On 14 dpi, enzyme immunoassay (EIA) was utilized to determine the antibody titer of hamster sera against SARS-CoV-2 NP antigen^40^. Briefly, 96-well immune-plates (Nunc) were coated with 100 μL/well (0.1 μg/well) of SARS-CoV-2 NP in 0.05 M NaHCO_3_ (pH 9.6) overnight at 4°C. After blocking, 100 μL of heat-inactivated serum samples were serial-diluted before adding to the wells and incubated at 37°C for 1 h. The attached antibodies were detected using horseradish-peroxidase-conjugated rabbit anti-hamster IgG antibody (Invitrogen, A18895). The reaction was developed by adding diluted 3,3’,5,5’-tetramethylbenzidine single solution (Invitrogen) and stopped with 0.3 N H_2_SO_4_. The optical density (OD) was read at 450/620 nm using a microplate reader.

### RNA-Seq analysis

Fastq files from RNA-seq were quality examined by FastQC (https://www.bioinformatics.babraham.ac.uk/projects/fastqc/). Reads were processed by cutadapt to remove reads with low quality and to trim adapters. Trimmed reads were aligned to hg38 reference genome using Tophat2^41^. Reads assigned to each gene were counted by featureCounts^42^ with refseq gene sets as references. Genes without at least 1 read mapped on average in each sample were considered undetectable and were filtered out. Read counts were normalized by Trimmed Mean of M-values (TMM) method and differential expression was calculated using R package edgeR and Genewise Negative Binomial Generalized Linear Models with Quasi-likelihood Tests (glmQLFit) method was used for statistical tests. Cut-offs imposed for differential expression analysis was set as False Discovery Rate (FDR) of 0.05 and fold change >2 or <0.5. The pathway analysis was performed by R package clusterProfiler^43^. Heatmaps were plotted using R package pheatmap (Kolde, R. (2013). pheatmap: Pretty Heatmaps. R package version 0.7.7. http://CRAN.R-project.org/package=pheatmap.). Other plots were generated by R package ggplot2 (Wickham H (2016). ggplot2: Elegant Graphics for Data Analysis. Springer-Verlag New York. ISBN 978-3-319-24277-4, https://ggplot2.tidyverse.org). The plots were made using Cytoscape^44^. PCA analysis was performed by R package factoextra.

### Pseudotyping of VSV and Pseudotypebased inhibition assay

Vesicular Stomatitis Virus (VSV) pseudotyped with spike proteins of MERS-CoV, SARS-CoV-1, and SARS-CoV-2 were generated as previously reported with some modifications^45^. Briefly, BHK-21/WI-2 cells (Kerafast, MA) overexpressing the spike proteins were inoculated with VSV-G pseudotyped AG-luciferase VSV (Kerafast, MA). After 2 h inoculation at 37°C, the inoculum was removed and cells were refed with DMEM supplemented with 5% FBS and VSV-G antibody (I1, mouse hybridoma supernatant from CRL-2700; ATCC). Pseudotyped particles were collected at 24 h post-inoculation, then centrifuged at 1,320 × g to remove cell debris and stored at −80°C until use.

To determine the effect of the compounds on viral entry, VeroE6 cells were treated with clofazimine at a concentration of 2.5 μM for 1 h prior to inoculation with respective pseudotyped VSV. After 2 h inoculation in the presence of the compounds, the inoculum was removed and cells were refed with fresh medium for further culture. The activity of firefly luciferase was measured using bright-Glo™ luciferase assay (Promega) for quantitative determination at 16 h post-transduction.

### The effect of clofazimine on SARSCoV-2 viral replication

The full-length SARS-CoV-2 viral RNA transcripts were *in vitro* synthesized from an infectious clone of SARS-CoV-2 (kindly provided by Pei-Yong Shi, UTMB) according to a recently published protocol^46^. 10 μg of total RNA transcripts and 5 μg SARS-CoV-2 NP gene transcript were mixed with VeroE6 cells stably expressing SARS-CoV-2 NP protein and then added into a 0.2 cm cuvette for nucleofection with the 4D-NucleofectorTM Core Unit (Lonza) using pulse code V-001. Immediately after electroporation, 1000 μL of pre-warmed media was added to the cuvette and cells were subsequently aliquoted into 384-well plates. Two hours post-seeding, compounds at different concentrations were added into each well. At 12 hours post-electroporation, intracellular and viral RNA was purified from the treated cells with TurboCapture 384 mRNA Kit (Qiagen) in accordance with the manufacturer’s instructions. The purified RNA was subjected to first-strand cDNA synthesis using the high-capacity cDNA reverse transcription kit (Applied Biosystems, Inc) with the following primer (TagRdRp-F: 5’- CGGT CAT GGTGG CGAATAACCCT GTGGGTTTTACACTTAA-3’). Real-time PCR analysis was performed using TaqPath 1-step RT-qPCR Master Mix (Applied Biosystems, Inc). The following primers and probe were used for negative-stranded RNA detection: Tag-F: 5’-CGGTCATGGTGGCGAATAACCCTGT-3’, ORF1ab-R: 5’-ACGATTGTGC ATCAGCTGA-3’, ORF1ab-P: 5’−6FAM-CCGTCTGCGGTATGTGGAAAGGTTATGG -BHQ1–3’).

### Timeof-addition assay

Time-of-drug-addition assay was performed to investigate which stage of SARS-CoV-2 life cycle clofazimine interfered with as previously described^47^. Briefly, VeroE6 cells were seeded in 96-well plates (4×10^4^ cells/well). The cells were infected by SARS-CoV-2 USA-WA1/2020 at an MOI of 1.5 and then incubated for additional 1 h. The viral inoculum was then removed and the cells were washed twice with PBS. At 1 hpi (i.e., post entry), clofazimine at a concentration of 5 μM was added to the infected cells at time-points indicated, followed by the incubation at 37 °C in 5% CO2 until 10 hpi (i.e. one virus life cycle). Cells were fixed at 10 hpi for quantification of the percentage of infected cells using an immunofluorescence assay targeting SARS-CoV-2 NP

## Supplementary Material

Supplement

Supplement
