# Supplementary figures and images for: Clofazimine is a broad-spectrum coronavirus inhibitor that antagonizes SARS-CoV-2 replication in primary human cell culture and hamsters
Source: Res Sq. 2020 Oct 7:rs.3.rs-86169. Preprint. [Version 1] doi: 10.21203/rs.3.rs-86169/v1 (PMC7553155; doi:10.21203/rs.3.rs-86169/v1)

# Extended Data Figure 2

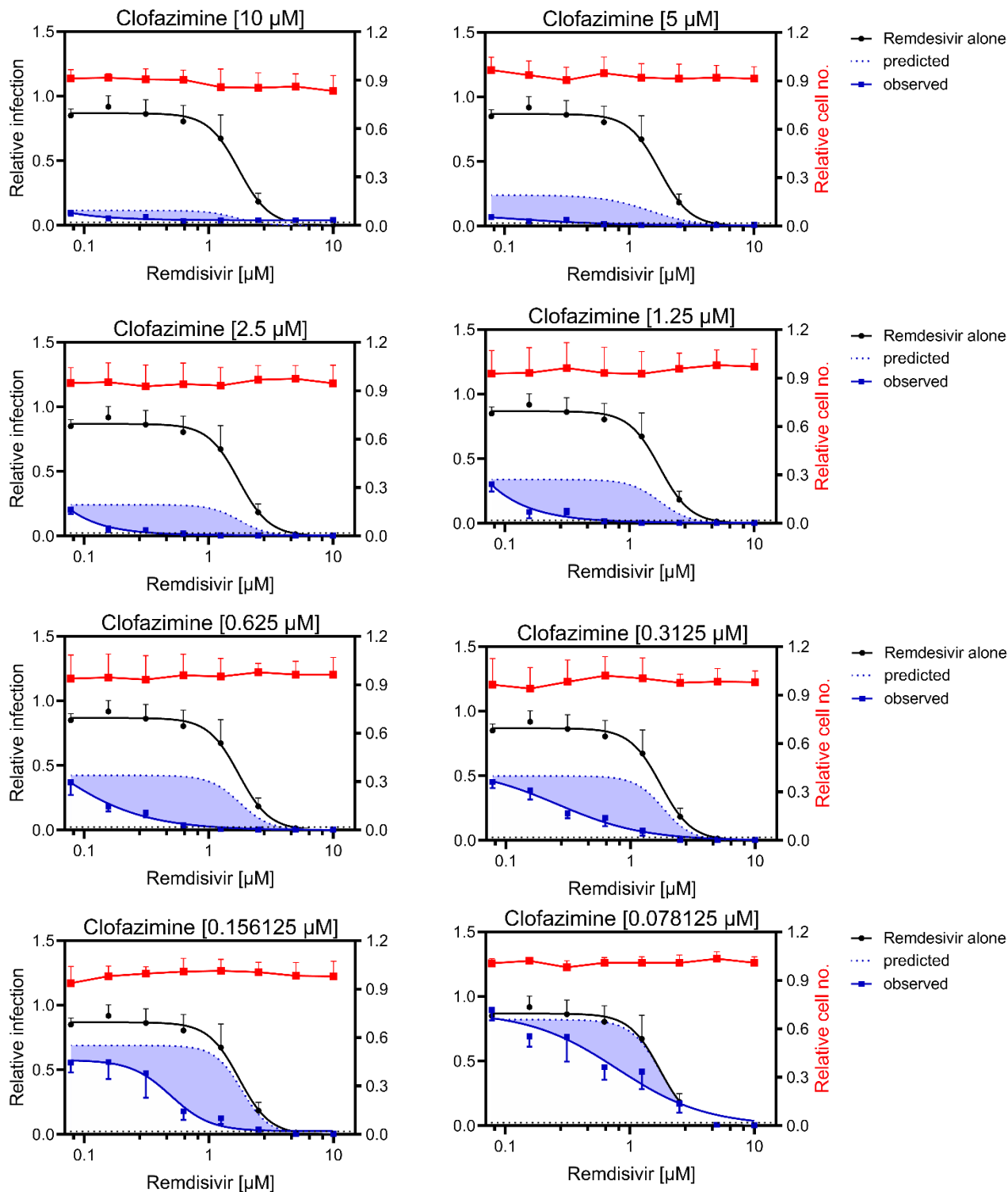

Supplement: Supplement [file 01dc9024c8fc4708b7e94310.pdf]

Extended Data Figure 1

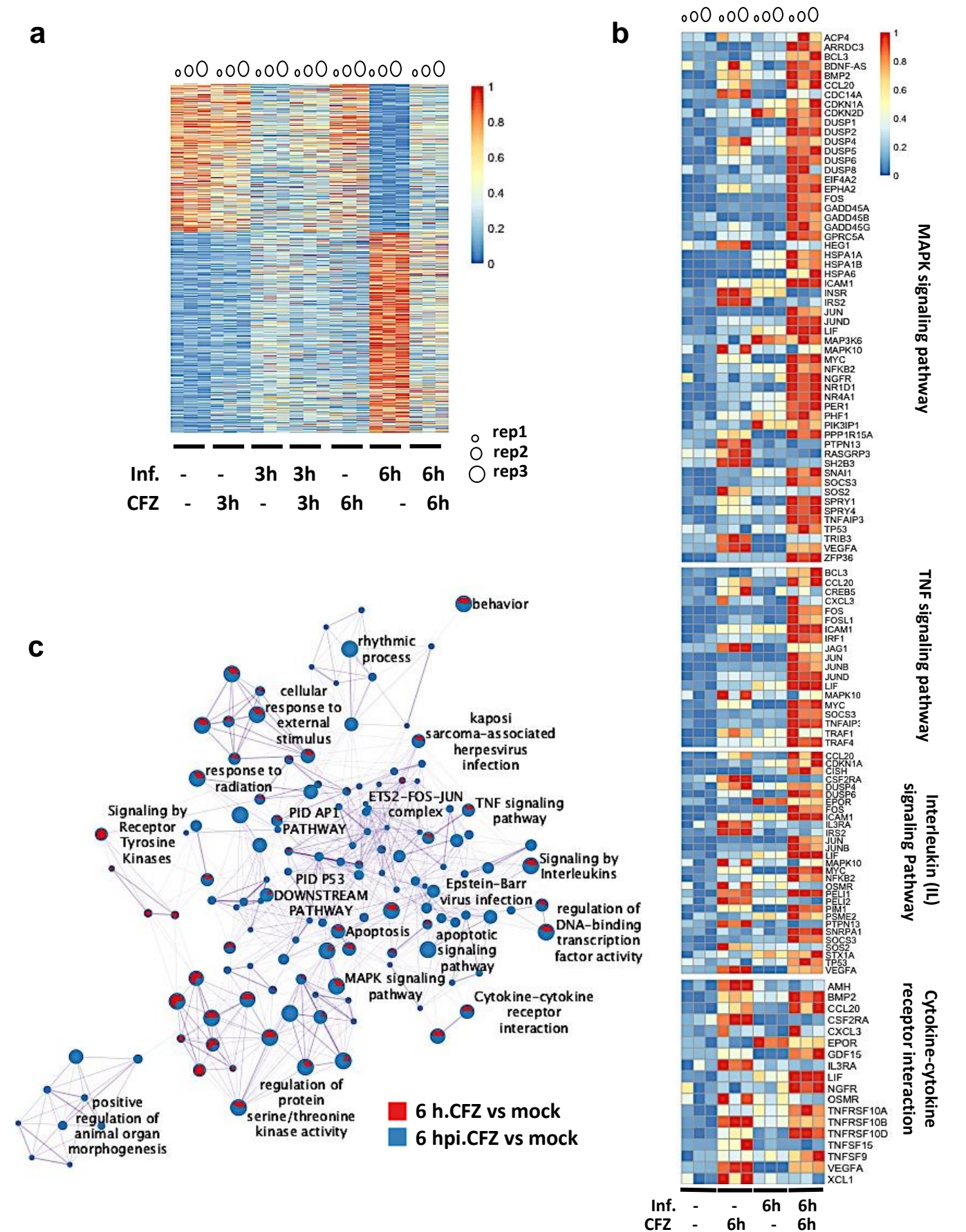

Supplement: Supplement [file f384fb5cad873e74fc9482a1.pdf]
